# Supplementary material for: Content-rich biological network constructed by mining PubMed abstracts
Source: BMC Bioinformatics. 2004 Oct 8;5:147. doi: 10.1186/1471-2105-5-147 (PMC528731; doi:10.1186/1471-2105-5-147)
Supplement: Additional File 2 — The original results of the above study (non-essential files are deleted to keep the file size under the limit set by BMC bioinformatics). [file 1471-2105-5-147-S2.bz2 › chilibotAdditionalFile2/dip50/450ID10477718E10461/html/CCL5_CCR5.html]

 


 **CCL5** and **CCR5** 
  
Found 460 abstracts in PubMed, retrieved 50.  
 

 What does Google say? 
 PDF only 
| .edu only 

---

**Interactive relationship** (e.g. stimulation, inhibition, etc)

**Inhibitory relationship**- Glycosaminoglycans removal from the cells by glycosaminidases treatment prevented RANTES  [ **CCL5** ]  binding to syndecan 1 and syndecan 4 and decreased RANTES  [ **CCL5** ]  binding to  **CCR5**  on the  **CCR5**  positive HeLa cells.  Ref: 12773479 Glycobiology< MedlineTA>Glycobiology,
- CONCLUSIONS Our results demonstrate that an interaction of HGV E2 with CD81 leads to increased RANTES  [ **CCL5** ]  secretion and decreased  **CCR5**  surface expression.  Ref: 12824783 AIDS, 2003
- The chemotactic response of  **CCR5**  CAM T82P to RANTES  [ **CCL5** ]  was similar to that of  **CCR5**  WT, but  **CCR5**  CAM T82K was dramatically decreased.  Ref: 12837756 J Biol Chem< MedlineTA>J Biol Chem, 2003
- None of the RANTES  [ **CCL5** ]  analogues caused internalization of a C terminal cytoplasmic domain deletion mutant of  **CCR5** , and each derivative had equal potency in inhibiting HIV 1 infection of MT 2 cells expressing this mutant.  Ref: 12543651 Antimicrob Agents Chemother, 2003
- Ad35K infected cell culture medium, containing recombinant 35K, potently reduced migration of  **CCR5**  transfected 293 cells by 95% in response to the CC chemokine RANTES  [ **CCL5** ] , but had no effect on cells transfected with the CX3CR1 fractalkine receptor.  Ref: 12777060 Immunobiology, 2003
- However, the pro adhesive and pro migratory capacities of SDF 1alpha and RANTES  [ **CCL5** ]  or MIP 1beta were mutually suppressed by the simultaneous or sequential exposure of the cells to these  **CCR5**  or CXCR4 ligands.  Ref: 12502723 Int Immunol, 2003
**Stimulatory relationship**- Whereas the amplitude of calcium mobilization induced by RANTES  [ **CCL5** ]  stimulation was lower in the  **CCR5**  CAM than the wild type WT receptor, MCP 1 induced a higher signal in the CCR2 CAM than in CCR2 WT.  Ref: 12837756 J Biol Chem< MedlineTA>J Biol Chem, 2003
- Therefore, RANTES  [ **CCL5** ]  bindings to syndecan 1 and syndecan 4 depend from glycosaminoglycans and facilitate RANTES  [ **CCL5** ]  interaction with  **CCR5** .  Ref: 12773479 Glycobiology< MedlineTA>Glycobiology, 2003
- Enhanced protein concentrations of RANTES  [ **CCL5** ] , MIP 1a, and MIP 1beta were present in WG lung lesions, indicating redundancy of ligands for  **CCR5**  in affected tissue.  Ref: 12858455 J Rheumatol, 2003
- The RANTES  [ **CCL5** ]  analogues showed the same rank order PSC > NNY > AOP in their capacity to induce prolonged  **CCR5**  internalization, inhibit surface reexpression, and prevent HIV 1 infection on MT 2 cells expressing wild type  **CCR5**  or  **CCR5**  with four C terminal serine phosphorylation sites mutated to alanine.  Ref: 12543651 Antimicrob Agents Chemother, 2003
**Neutral relationship**- This complex is likely a functional unit in which PGs may modulate RANTES  [ **CCL5** ]  binding to  **CCR5** .  Ref: 12773479 Glycobiology< MedlineTA>Glycobiology, 2003
- P2 resembles AOP RANTES  [ **CCL5** ]  in that it is a superagonist of  **CCR5**  and potently induces receptor sequestration.  Ref: 12767983 J Virol, 2003
- We conclude that the C terminal cytoplasmic residues of  **CCR5**  are necessary for receptor sequestration by RANTES  [ **CCL5** ]  analogues but that the process and the relative activity of each derivative are not dependent upon phosphorylation of the C terminal serine residues.  Ref: 12543651 Antimicrob Agents Chemother, 2003
- Confocal microscopy analysis as well as co immunoprecipitation experiments show a RANTES  [ **CCL5** ]  independent heteromeric complex, which comprises on the  **CCR5**  positive HeLa cells, syndecan 1, syndecan 4 and  **CCR5** .  Ref: 12773479 Glycobiology< MedlineTA>Glycobiology, 2003
- **CCR5**  is a G protein coupled receptor for RANTES  [ **CCL5** ] , MIP 1a, MIP 1b and MCP 2 that functions as the front line coreceptor for human immunodeficiency virus type 1 infection.  Ref: 12837756 J Biol Chem< MedlineTA>J Biol Chem, 2003
- C C chemokine receptor 5  [ **CCR5** ]  is receptor for the C C chemokine RANTES  [ **CCL5** ] , which is expressed in inflammatory kidney diseases.  Ref: 12600179 Pol Arch Med Wewn, 2002
- IL 8 activation of CXCR1, but not the IL 8 receptor CXCR2, cross phosphorylated  **CCR5**  and CXCR4 and cross desensitized their responsiveness to RANTES  [ **CCL5** ]  regulated on activation normal T cell expressed and secreted  **CCL5**  and stromal derived factor SDF 1 or CXCL12, respectively.  Ref: 12594210 J Biol Chem, 2003
- However, the remaining expression of CCR1 and  **CCR5**  correlated reciprocally with an ongoing unresponsiveness to the CCR1 and  **CCR5**  agonists CCL3 macrophage inflammatory protein 1alpha MIP 1alpha, CCL4 MIP 1beta, and  **CCL5**  regulated on activation, normal T expressed and secreted RANTES  [ **CCL5** ] , all chemokines binding to these two receptors.  Ref: 12885942 J Leukoc Biol, 2003
- Co immunoprecipitation studies demonstrate that RANTES  [ **CCL5** ]  forms complexes with glycanated syndecan 1 and syndecan 4, in addition to  **CCR5**  on the  **CCR5**  positive HeLa cells.  Ref: 12773479 Glycobiology< MedlineTA>Glycobiology, 2003
- However, the specific functional relevance of RANTES  [ **CCL5** ]  oligomerization for distinct steps of leukocyte recruitment on inflamed endothelium mediated by the RANTES  [ **CCL5** ]  receptors CCR1 and  **CCR5**  remains undefined.  Ref: 12763925 Blood< MedlineTA>Blood, 2003
- This interaction induces a dose dependent release of RANTES  [ **CCL5** ]  and down regulation of  **CCR5**  surface expression with concomitant intra cellular accumulation of  **CCR5**  proteins.  Ref: 12824783 AIDS, 2003
- The present findings suggest that MCP 1, RANTES  [ **CCL5** ] , and IP 10 may participate in the pathogenesis of mercuric chloride induced tubulointerstitial fibrosis with mononuclear cell infiltration, via CCR2, CCR1 or  **CCR5** , and CXCR3, respectively.  Ref: 12834626 Exp Mol Pathol, 2003
- Our studies showed that LD infection caused the upregulation of three beta chemokines macrophage inflammatory protein 1 alpha MIP 1alpha, MIP 1beta and RANTES  [ **CCL5** ]  regulated on activation normal T cell expressed and secreted, one alpha chemokine interleukin 8 IL 8 and the CC chemokine receptor 5  [ **CCR5** ]   **CCR5**  but not CCR1, as evident from reverse transcriptase polymerase chain reaction RT PCR analysis.  Ref: 12662300 Scand J Immunol, 2003

**Non-interactive relationship** (e.g. studied together, co-existance, homology, etc.)

- Only CpG ODN exacerbated HAF GN with an increase of glomerular macrophages, which was associated with massive albuminuria and increased renal MCP 1 CCL2, RANTES  [ **CCL5** ]   **CCL5** , CCR1, CCR2, and  **CCR5**  mRNA expression.  Ref: 12538732 J Am Soc Nephrol, 2003
- The  **CCR5**  ligands, RANTES  [ **CCL5** ]  and MIP 1 alpha, are expressed in the spinal cords of mice at EAE onset.  Ref: 12667654 J Neuroimmunol, 2003
- RESULTS Expression of IP 10, Mig, I TAC, RANTES  [ **CCL5** ] , CXCR3, and  **CCR5** , but not MCP 1 and IL 8, increased significantly in both grade 2 and grade 3 rejection P< ; =0.0096.  Ref: 12544874 Transplantation, 2003
- KA treatment did not affect mRNA levels for the macrophage inflammatory protein 1alpha MIP 1alpha or the regulated upon activation normal T cells expressed and secreted protein RANTES  [ **CCL5** ]  in either wild type or  **CCR5**  KO mice.  Ref: 12807425 J Neurochem, 2003
- Discriminant analysis showed that the RANTES  [ **CCL5** ]  28G positive genotype and  **CCR5**  59029A positive genotype were independently associated with nephropathy.  Ref: 12610055 Diabetes Care, 2003
- CONCLUSIONS The RANTES  [ **CCL5** ]  promoter 28G genotype and  **CCR5**  promoter 59029A genotype may be independent risk factors for diabetic nephropathy in patients with type 2 diabetes and may have an additive effect on nephropathy.  Ref: 12610055 Diabetes Care, 2003
- Matching pairs of receptors ligands were found to be coordinately expressed, including TGFbetaRII with TGFbeta3, FGFRII and cysteine rich fibroblast growth factor receptor CRF 1 with FGF7 and FGF12, CCR1, CCR3,  **CCR5**  with RANTES  [ **CCL5** ]  and calcitronin receptor like gene CALCRL with adrenomedullin.  Ref: 12584245 J Cell Sci, 2003
- Potential molecular differences between early and late rejection grade > =2 episodes were examined by testing chemokine and receptor gene expression.METHODS Expression of inducible protein IP 10, monokine induced by IFN gamma Mig, interferon inducible T cell alpha chemoattractant I TAC, regulated on activation normal T cell expressed and secreted RANTES  [ **CCL5** ] , and their receptors CXCR3 and  **CCR5**  was tested in 60 endomyocardial biopsies from 24 patients using quantitative Taqman real time polymerase chain reaction PCR.  Ref: 12829909 Transplantation, 2003
- Polymorphisms of 28 C G and 403 G A in the RANTES  [ **CCL5** ]  gene promoter region, and of 59029 G A in the  **CCR5**  gene promoter region were detected by PCR RFLP restriction fragment length polymorphism.  Ref: 12610055 Diabetes Care, 2003
- In parallel, CCR1 and  **CCR5**  expression is down regulated and CXCL8, CCL2, CCL3 and  **CCL5**  production is up regulated.  Ref: 12548563 Eur J Immunol, 2003
- there was little effect on leukocyte infiltration in xenografts harvested from  **CCR5**  mice lacking the co receptor for MIP 1alpha, MIP 1beta and RANTES  [ **CCL5** ] .  Ref: 12588649 Xenotransplantation, 2003
- In contrast, the loss of the  **CCR5**  ligand, CCL3, led to an early delay in tumor growth that did not persist, while the absence of the  **CCR5**  ligand,  **CCL5** , had no effect.  Ref: 12682253 J Immunol, 2003
- P1, while less potent than P2, has the advantage of significantly reduced signaling activity via  **CCR5**  30% of that of RANTES  [ **CCL5** ] .  Ref: 12767983 J Virol, 2003
- After 2 to 4 weeks, L. amazonensis infected mice had significantly delayed and depressed expression of inflammatory cytokines interleukin 12 IL 12, gamma interferon, IL 1alpha, IL 1beta, CC chemokines CC chemokine ligand 3 CCL3 macrophage inflammatory protein 1alpha MIP 1alpha, CCL4 MIP 1beta,  **CCL5**  RANTES  [ **CCL5** ] , MIP 2, and chemokine receptors CCR1, CCR2,  **CCR5**  in foot tissues and draining lymph nodes compared to the expression in L. major infected controls.  Ref: 12874303 Infect Immun, 2003
- Oligomerization of RANTES  [ **CCL5** ]  is required for CCR1 mediated arrest but not  **CCR5**  mediated transmigration of leukocytes on inflamed endothelium.  Ref: 12763925 Blood< MedlineTA>Blood, 2003
- Infiltrating CD8 T Cells in Oral Lichen Planus Predominantly Express  **CCR5**  and CXCR3 and Carry Respective Chemokine Ligands RANTES  [ **CCL5** ]   **CCL5**  and IP 10 CXCL10 in Their Cytolytic Granules A Potential Self Recruiting Mechanism.  Ref: 12819030 Am J Pathol, 2003
- To test the in vivo relevance of this, we used a murine melanoma system and knockout mice to investigate the function of the chemokine receptor  **CCR5**  and its ligands, CCR ligand CCL 3 and  **CCL5** .  Ref: 12682253 J Immunol, 2003
- Furthermore, infiltrating T cells, particularly CD8 T cells, were positive for RANTES  [ **CCL5** ]   **CCL5**  and IP 10 CXCL10, the ligands of  **CCR5**  and CXCR3, respectively.  Ref: 12819030 Am J Pathol, 2003
- Strikingly, early CXCR4 signaling of phosphatidylinositol 3 kinase, detected by SDF 1alpha induced AKT phosphorylation, was insensitive to RANTES  [ **CCL5** ]   **CCR5**  signals.  Ref: 12502723 Int Immunol, 2003
- Removal of glycosaminoglycans by glycosaminidases treatment of the complexes, RANTES  [ **CCL5** ]  syndecan 1 syndecan 4  **CCR5** , immobilized on beads, reversed syndecan 1 and syndecan 4 bindings.  Ref: 12773479 Glycobiology< MedlineTA>Glycobiology, 2003
- **CCR5**  D32 and RANTES  [ **CCL5** ]  28 and 401 polymorphisms were determined by either specific primers or direct sequencing.  Ref: 12858455 J Rheumatol, 2003
- In the present study, we examined the expression of chemokine receptors CXCR1 and CXCR2 receptors for interleukin 8 and  **CCR5**  receptor for RANTES  [ **CCL5** ]  regulated on activation, normal T cell expressed and secreted, macrophage inflammatory protein MIP 1alpha, and MIP 1beta in human endometrium.  Ref: 12606476 Biol Reprod, 2003
- METHODS Lung biopsies from 4 patients that had classical features of WG were examined for protein expression of  **CCR5** , RANTES  [ **CCL5** ] , MIP 1a and MIP 1beta using immunohistochemistry.  Ref: 12858455 J Rheumatol, 2003
- This structure of V3 MN was found to have conformation and sequence similarities to beta hairpins in CD8 and  **CCR5**  ligands MIP 1alpha, MIP 1beta, and RANTES  [ **CCL5** ]  and differed from the beta hairpin of a V3 IIIB peptide bound to the strain specific murine anti gp120 IIIB antibody 0.5beta.  Ref: 12575942 Structure Camb , 2003
- The beta chemokines RANTES  [ **CCL5** ]  regulated on activation, normal T cell expressed and secreted, macrophage inflammatory protein 1alpha MIP 1alpha, and MIP 1beta are the natural ligands of the HIV 1 coreceptor  **CCR5**  and compete with the virus for receptor binding.  Ref: 12644703 Proc Natl Acad Sci U S A, 2003
- We have investigated the inhibitory mechanism of three N terminally modified RANTES  [ **CCL5** ]  variants AOP, NNY, and PSC RANTES  [ **CCL5** ]  with the MT 2 human T cell line stably expressing either native or mutated  **CCR5** .  Ref: 12543651 Antimicrob Agents Chemother, 2003
- We examined the responses of human peripheral blood T cells, present in an ECM like context, to combinatorial signaling transduced by SDF 1alpha CXCL12, and two  **CCR5**  ligands, RANTES  [ **CCL5** ]   **CCL5**  and MIP 1beta CCL4.  Ref: 12502723 Int Immunol, 2003
- The frequency of a RANTES  [ **CCL5** ]  28G positive genotype C G or G G was higher in the DN2 group than in the DN0 and DN1 groups 34% vs. 25 and 20%, P = 0.0268, chi 2 = 4.905, and the frequency of a  **CCR5**  59029 A positive genotype G A or A A was higher in the DN1 and DN2 groups than in the DN0 group 84 and 85% vs. 76%, P = 0.0123, chi 2 = 6.269.  Ref: 12610055 Diabetes Care, 2003
- Notably, the formation of higher order RANTES  [ **CCL5** ]  oligomers was crucial for CCR1 mediated arrest but not for  **CCR5**  mediated spreading transmigration in flow or transendothelial chemotaxis of leukocytes.  Ref: 12763925 Blood< MedlineTA>Blood, 2003
- OBJECTIVE To evaluate the effect of RANTES  [ **CCL5** ]  gene promoter polymorphism and RANTES  [ **CCL5** ]  receptor  **CCR5**  gene promoter polymorphism on diabetic nephropathy in Japanese type 2 diabetic subjects.  Ref: 12610055 Diabetes Care, 2003
- CONCLUSION The expression of IP 10, Mig, I TAC, RANTES  [ **CCL5** ] , CXCR3, and  **CCR5**  in cardiac allografts significantly correlates with the presence and grade of acute rejection.  Ref: 12544874 Transplantation, 2003
- A SHIV strain termed SHIV JRFL could enter cells via CD4 with a chemokine receptor  **CCR5** , not CXCR4, and the viral replication was suppressed by recombinant human RANTES  [ **CCL5** ] , one of beta chemokines.  Ref: 12655130 J Vet Med Sci, 2003
- Expression of  **CCR5**  and RANTES  [ **CCL5** ]  may be important in the modulation of hepatic inflammation and response to interferon therapy in chronic hepatitis C.  Ref: 12557141 Gastroenterology, 2003
- Furthermore, the presence of  **CCL5**  and CXCL10 in the cytolytic granules of tissue infiltrating CD8 T cells expressing  **CCR5**  and CXCR3 reveals a potential self recruiting mechanism involving activated effector cytotoxic T cells.  Ref: 12819030 Am J Pathol, 2003
- Furthermore, aminoxypentane RANTES  [ **CCL5** ]  abolishes virus replication, suggesting that these SIV strains utilize the chemokine receptor  **CCR5**  for entry into astrocytes.  Ref: 12768005 J Virol, 2003
- We show that the CC chemokine, RANTES  [ **CCL5** ] , binds not only to high affinity binding sites on  **CCR5**  positive HeLa cells, but also to low affinity binding sites on HeLa cells, expressing or lacking RANTES  [ **CCL5** ]  G protein coupled receptors.  Ref: 12773479 Glycobiology< MedlineTA>Glycobiology, 2003
- All 24 R5 isolates were inhibited by the  **CCR5**  specific agents, PRO 140 and RANTES  [ **CCL5** ] , while the two X4 viruses and the three R5X4 viruses were sensitive to the CXCR4 specific inhibitor, AMD3100.  Ref: 12634405 J Virol, 2003
- METHODS Six chemokine system polymorphisms CCR5Delta32,  **CCR5**  promoter 59029 G A, CCR2 64I, RANTES  [ **CCL5** ]  regulated upon activation, normal T cells expressed and secreted 403 G A, and 28 C G and stromal derived factor 1 3 A were studied in 417 patients with liver diseases 339 with hepatitis C and 2380 blood donors.  Ref: 12557141 Gastroenterology, 2003
- In conclusion, RANTES  [ **CCL5** ]  and  **CCR5**  represent potential mediators of 1 HSC migration and proliferation and 2 a cross talk between HSCs and leukocytes during fibrogenesis.  Ref: 12829440 Am J Physiol Gastrointest Liver Physiol< MedlineTA>Am J Physiol Gastrointest Liver Physiol, 2003
- Human Hepatic Stellate Cells express  **CCR5**  and RANTES  [ **CCL5** ]  to induce proliferation and migration.  Ref: 12829440 Am J Physiol Gastrointest Liver Physiol< MedlineTA>Am J Physiol Gastrointest Liver Physiol, 2003
- The expression of interferon IFN gamma inducible protein IP 10, monokine induced by IFN gamma Mig, interferon inducible T cell alpha chemoattractant I TAC, regulated on activation normal T cell expressed and secreted RANTES  [ **CCL5** ] , monocyte chemotactic protein MCP 1, interleukin IL 8, and the receptors CXCR3 and  **CCR5**  were tested using quantitative, real time polymerase chain reaction.  Ref: 12544874 Transplantation, 2003
- In J774 cells, CpG ODN induced the chemokines MCP 1 CCL2 and RANTES  [ **CCL5** ]   **CCL5**  and the chemokine receptors CCR1 and  **CCR5** .  Ref: 12538732 J Am Soc Nephrol, 2003
- The chemokine CC chemokine ligand CCL 5  [ **CCL5** ]  RANTES  [ **CCL5** ]  as well as its respective receptor  **CCR5**  mediate leukocyte infiltration during inflammation and are up regulated early during the course of glomerulonephritis GN.  Ref: 12759447 J Immunol, 2003
- The biopsies were taken in the first 3 months or from the 9th to the 12th month following transplantation.RESULTS IP 10, Mig, RANTES  [ **CCL5** ] , CXCR3, and  **CCR5**  expression levels were increased in the later versus earlier biopsies P< ; =0.01 despite no change in histologic rejection grade status.CONCLUSION These results demonstrate significantly increased expression of T cell chemoattractants in heart allografts during later rejection when compared with episodes occurring shortly after transplantation.  Ref: 12829909 Transplantation, 2003
- As well as potently inhibiting the entry of  **CCR5**  using HIV 1 strains, RANTES  [ **CCL5** ]  can enhance HIV 1 infectivity under certain experimental conditions.  Ref: 12714503 Blood< MedlineTA>Blood, 2003
